# Structured neurological soft signs examination reveals motor coordination deficits in adults diagnosed with high-functioning autism

**DOI:** 10.1038/s41598-024-66723-5

**Published:** 2024-07-12

**Authors:** Jelte Wieting, Madita Vanessa Baumann, Stephanie Deest-Gaubatz, Stefan Bleich, Christian Karl Eberlein, Helge Frieling, Maximilian Deest

**Affiliations:** 1 Hannover Medical School, Department of Psychiatry, Social Psychiatry and Psychotherapy, Carl-Neuberg-Str. 1, 30625 Hannover, Germany; 2 Oberberg Fachklinik Weserbergland, Brede 29, 32699 Extertal-Laßbruch, Germany

**Keywords:** High functioning autism, Autism spectrum disease, Neurological soft signs, Motor coordination, Adult, Intelligence, Autism spectrum disorders, Neurological manifestations, Diagnostic markers

## Abstract

Neurological soft signs (NSS), discrete deficits in motor coordination and sensory integration, have shown promise as markers in autism diagnosis. While motor impairments, partly associated with core behavioral features, are frequently found in children with autism, there is limited evidence in adults. In this study, NSS were assessed in adults undergoing initial diagnosis of high-functioning autism (HFA), a subgroup difficult to diagnose due to social adaptation and psychiatric comorbidity. Adults with HFA (n = 34) and 1:1 sex-, age-, and intelligence-matched neurotypical controls were administered a structured NSS examination including motor, sensory, and visuospatial tasks. We showed that adults with HFA have significantly increased motor coordination deficits compared with controls. Using hierarchical cluster analysis within the HFA group, we also identified a subgroup that was particularly highly affected by NSS. This subgroup differed from the less affected by intelligence level, but not severity of autism behavioral features nor global psychological distress. It remains questionable whether motor impairment represents a genuinely autistic trait or is more a consequence of factors such as intelligence. Nevertheless, we conclude that examining NSS in terms of motor coordination may help diagnose adults with HFA and identify HFA individuals who might benefit from motor skills interventions.

## Introduction

Autism spectrum disorder (ASD) is characterized by persistent deficits in social interaction and communication and restrictive, repetitive behavioral patterns with onset typically during childhood. In addition, children with ASD were frequently shown to display motor impairments of so far not conclusively understood origin^1,2^. Furthermore, motor impairments have been shown to correlate with core behavioral features of ASD, such as social interaction and communication deficits^3^. Schilbach conceptualized psychiatric disorders, including ASD, as "disorders of social interaction" that result from impairments in the ability to engage in successful social interactions^4^. To this end, it should be noted that successful social interactions require the integration of various cognitive skills, including action observation, social cue perception, and theory of mind, as well as motor skills, i.e., the ability to produce appropriate motor behaviors, such as gestures, facial expressions, and coordinated movements, that are critical for successful social exchange. This has led to the ongoing debate about the inclusion of motor development deficits in the diagnostic criteria for ASD. In a recent meta-analysis Wang et al. demonstrated an overall deficit in gross motor skills in children with ASD accompanied by a modest correlation between motor and social skills^5^.

However, in high-functioning forms of autism spectrum disorders (HFA) symptoms may not become fully manifest until adulthood. Due to the high prevalence of psychiatric comorbidities, but also a partly high degree of adaptation to social requirements, the diagnosis of HFA in adulthood is difficult and a clinical challenge. Hence, only few data can be found regarding autism associated motor impairments in adults, especially in HFA. Adults with autism significantly more often self-reported a co-diagnosis of dyspraxia^6^. A recent study on motor ability in HFA provided hints towards specific neuro-motor signatures affecting timing and balance in HFA adults^7^.

As previous studies described autism-related dyspraxia to be driven by motor coordination dysfunction and impaired visual-motor integration^8^, a structured examination of neurological soft signs (NSS) appeared as a promising option in detecting autism-associated motor impairment. NSS are originally defined as discrete, non-localizable deficits in motor coordination, sequencing of complex motor tasks and sensory integration^9^. Usually initially observed during childhood, persistence of NSS to adulthood is described to be associated with an increased risk of psychiatric disorders. This led to the neurodevelopmental hypothesis of NSS with discrete neurological deficits as signs of impaired brain development and markers of vulnerability for neurodevelopmental disorders (NDD)^10^. NSS are evidenced across a variety of conditions, pointing towards a transdiagnostic neurodevelopmental impairment in psychiatric diseases^11^. As they were additionally frequently found to be independent of demographic and treatment variables, NSS emerged as an overall promising tool in psychiatric diagnosis^12,13^.

Though ASD being one of the most prevalent NDD, it is sparsely investigated regarding structured NSS examination, especially in adults with HFA. Mayoral et al. found HFA to be associated with increased NSS in children and adolescents^14^. Hirjak et al. examined NSS in young adults with HFA compared to healthy control subjects and found a significantly elevated global NSS score in the HFA group^15^. Consistently, Tani et al. found globally increased NSS scores in a small sample of young adults with HFA^16^. Both studies suggested NSS in HFA to persist to adulthood.

In summary, previous studies provided first hints towards NSS as promising additional markers in diagnosis of HFA in adults. However, those studies excluded or negated psychiatric comorbidity, which barely reflects diagnostic reality given the high prevalence of psychiatric comorbidities in adults with HFA^17,18^. At least in children with HFA, an association of NSS with psychiatric comorbidities has been described^19^. Moreover, previous studies on NSS in adult HFA do not address the question to what extent there is an association between NSS and autism-typical behavioral features as found between motor and social skills in autistic children^5^. Furthermore, the question arises whether NSS might affect only a certain subgroup of adults with HFA, and how this subgroup might differ from the norm.

The present study was designed to broadly examine NSS in adults undergoing first-time diagnosis of HFA using the Neurological Soft Sign Examination Scale comprising motor, sensory, and visuospatial tasks. First, NSS outcomes of subjects diagnosed with HFA in adulthood were compared to sex-, age and intelligence quotient (IQ)-matched healthy controls. In a second step, we investigated whether subjects with HFA could be statistically classified into natural groups based on their NSS scores and which demographic and clinical variables characterize these groups. We expected this approach to provide additional information about the potential of structured NSS examination as an additional marker in diagnosis of HFA in adulthood.

## Results

### Demographics

Total number of subjects was N = 75. In HFA group, n = 3 subjects were manually excluded for potentially confounding disease (hip dysplasia / Perthes’ disease, essential tremor and epilepsy) as well as n = 3 HFA and n = 1 control subjects for missing data. Matching for sex, age and IQ resulted in n = 29 HFA-control pairs (n total = 58, 24 male, 34 female); both in HFA and control subjects n = 5 were excluded for missing match. After matching, mean age (32.34 ± 9.08 y in HFA, 32.14 ± 9.841 in controls, p = 0.741) and IQ total (108.03 ± 9.90 in HFA, 109.72 ± 10.18 in controls, p = 0.2611) differed insignificantly between groups. In HFA group 51.7% stated to be regularly treated with antidepressant and 13.8% with neuroleptic medication (with n = 8 missing information on medical history). Table 1 provides a detailed demographic overview.

**Table 1:** Demographics of HFA compared to sex-, age- and IQ-matched controls (pairs matched 1:1 with sex exact, age ± 5 years and IQ total ± 15 points).

	Matched pairs *(HFA and control)*				
n =	29				
Sex	12 male (41.4%)				
	17 female (58.6%)				

		Mean	Minimum	Maximum	SD
Age	HFA	32.34	20	52	9.076
	Controls	32.14	20	55	9.841
IQ total	HFA	108.03	88	125	9.898
	Controls	109.72	82	131	10.184

	Comparison of groups			p	
	Mean difference	SD	95% CI		
Age (in y)	− 0.207	3.342	− 1.478 to 1.064	0.741	
IQ total (points)	1.690	7.933	− 1.328 to 4.707	0.261	

HFA subjects excluded for missing match in the first step, were included for cluster analysis (n = 34).

### Group comparison

The overall comparison of NSS total scores by group showed no significant difference between HFA and controls in the average of all individual items combined (0.684 ± 0.242 for HFA, 0.632 ± 0.191 for controls, p = 0.412), nor in the average of the subscale scores combined (0.522 ± 0.223 for HFA, 0.475 ± 0.189 for controls, p = 0.400).

**Figure 1:**
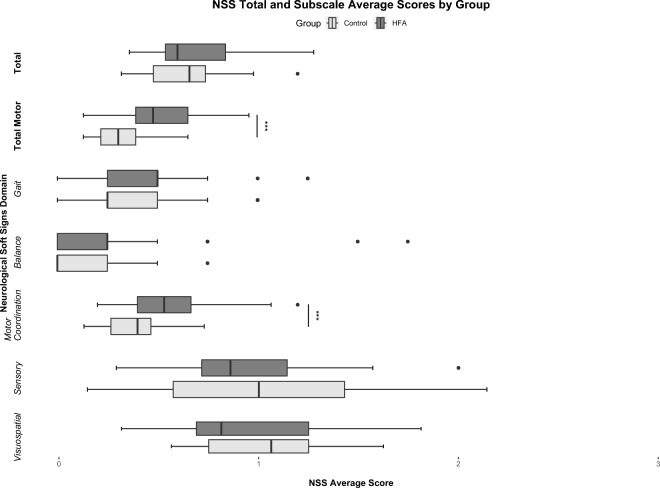
NSS total and subscale scores (average scores per item, 0–3) compared between HFA and controls. While there is no group difference in total average scores, HFA tend to score significantly higher in motor function related subscales (gait, balance, motor coordination) combined within the total motor scale, but when looking at the individual subscales with significant difference between groups only in the NSS subscale motor coordination, p < 0.001. The motor subscale displays lower and upper extremity motor coordination skills (see Supplementary Material S1). Especially in HFA group, extreme outliers regarding NSS gait and balance scores are evident (closed circles). Contrary, in sensory and visuospatial tasks HFA tend to score even lower than controls, but without statistical significant group differences.

Comparison of NSS subscales, however, revealed a significant group difference regarding subscale motor coordination, t (56) = 3.936, p adj. < 0.001, with an average score of 0.584 ± 0.046 in HFA and 0.368 ± 0.030 in controls, displaying a large effect according to Cohen at d = 1.034. Initially, also NSS subscale gait appeared to differ significantly between groups with average scores of 0,466 ± 0.062 in HFA and 0.319 ± 0.055 in controls, U = 297.500, Z = -1.990, p = 0.047. However, p did not stay significant with post-hoc Bonferroni-Holm correction, p adj. = 0.282. NSS subscale balance also tended to increased average scores in HFA (0.302 ± 0.080 to 0.129 ± 0.036 in controls), but without significant group differences, p adj. = 0.875. Also, no significant differences were seen for subscales sensory perception, p adj. =  > 0.999, and visuospatial function, p adj. =  > 0.999. Contrary to the other subscales, both average scores trended to be decreased in HFA compared to controls with average NSS scores of 0.941 ± 0.072 to 0.961 ± 0.092 in subscale sensory perception and 0.951 ± 0.073 to 1.048 ± 0.056 in visuospatial function. On motor-associated subscales gait and balance, the HFA group showed partly extreme outliers towards higher average scores. Figure 1 gives a graphical representation of the group comparison, Table 2 provides a tabular overview of the statistical results.

**Table 2:**
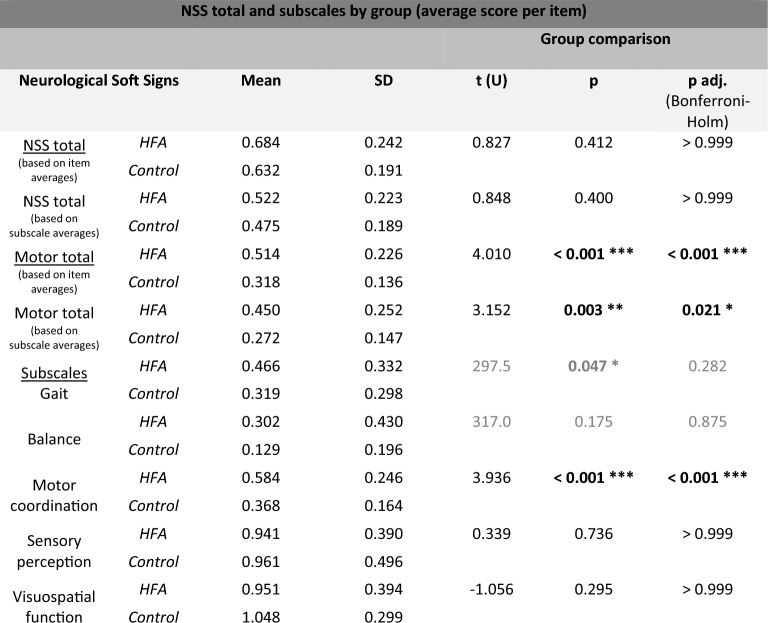
NSS total and subscale scores compared by group HFA versus healthy controls (t tests or non-parametric Mann–Whitney tests were applied depending on distribution of data; scales tested by Mann–Whitney-U test are grayed).

Significant values are in bold.

Subscale motor coordination differed highly significant between groups after correction for multiple testing while comparison regarding the NSS total scale remained insignificant. However, the total motor scale, which combines the motor subscales of gait, balance and motor coordination, also showed significant group differences.

*p < 0.05, **p < 0.01, ***p < 0.001.

NSS subscales reflex (including solely palmomental reflex item) and other (two items non fitting elsewhere) were removed from further analysis as averages strongly trended to zero with few extreme outliers.

As motor associated subscales were shown to trend towards higher average scores in HFA opposite to the remaining scales, we further combined the respective subscales gait, balance and motor coordination to an average score comprising all three motor associated scales (individual items considered equally). With an average of 0.514 ± 0.226 in HFA compared to 0.318 ± 0.136 in controls, group comparison of the resulting “total motor” scale showed a highly significant group difference, t (45.811) = 4.010, p < 0.001, displaying a large effect according to Cohen, d = 1.053. This effect is also discernible in a diminished form when the average values of the three subscales are considered in an equal manner (HFA 0.450 ± 0.252, controls 0.272 ± 0.147, p = 0.003).

In addition, an analysis of NSS data by sex was performed within the HFA group. Thereby, the sexes differed only, but highly significant, regarding the NSS subscale sensory perception (males 1.202 ± 0.424, females 0.756 ± 0.236, p adj. = 0.030). That difference had no effect on the entire NSS scale (males 0.750 ± 0.251, females 0.638 ± 0.232, p adj. > 0.999). If controls were included, the effect regarding sensory subscale persisted (males 1.232 ± 0.416, females 0.752 ± 0.345, p adj. < 0.001), but was accompanied by a slightly significant difference on the NSS total scale (males 0.748 ± 0.228, females 0.595 ± 0.189, p adj. = 0.035). The remaining scales showed no significant differences when compared by sex (data not shown).

### Cluster analysis

**Figure 2:**
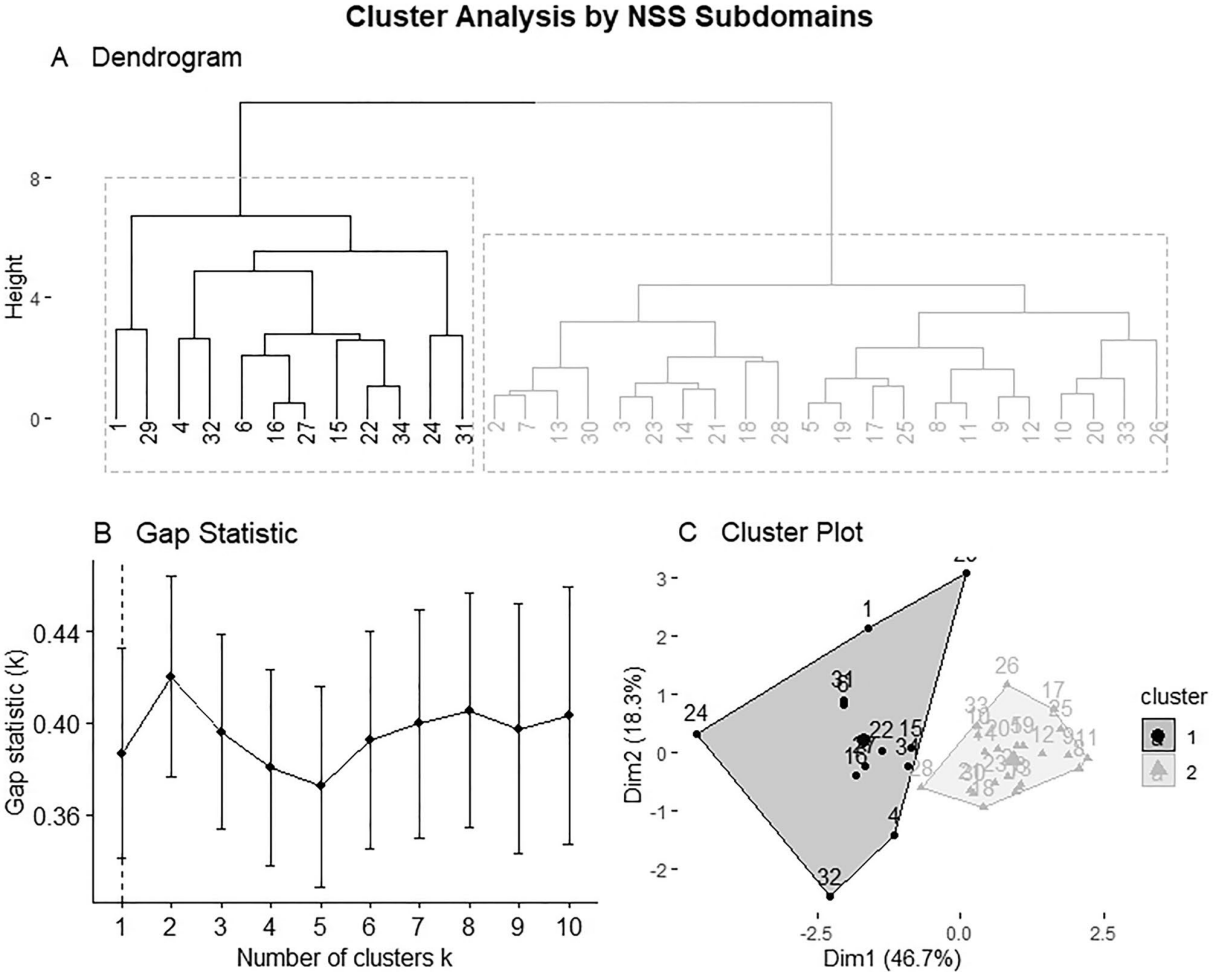
Dendrogram showing the hierarchical clustering process within HFA group based on the scores of NSS subscales gait, balance, motor coordination, sensory perception and visuospatial function. HFA Subjects were stepwise combined into clusters minimizing the within-cluster variance (Ward’s method). The y-axis gives a measure of heterogeneity, whereas the x-axis gives the IDs of the individual HFA subjects examined (n = 34) (**A**). The appropriate number of clusters can be determined based on the largest increase in heterogeneity. In this case, a two-cluster solution is the obvious choice, which is supported by the gap statistic (**B**), showing its maximum at k = 2. The cluster plot based on hierarchical clustering (**C**) shows two separated clusters within HFA group, further referred to as cluster 1 (n = 12, dark grey) and cluster 2 (n = 22, light grey). Within Cluster 1, an increased variance between data points is visually evident.

Figure 2 shows the hierarchical clustering process within HFA group based on the scores of NSS subscales gait, balance, motor coordination, sensory perception and visuospatial function. HFA subjects were stepwise combined into clusters minimizing the within-cluster variance as represented in Fig. 2A. The largest increase in heterogeneity was seen with a two-cluster solution (Fig. 2B). The cluster plot based on hierarchical clustering (Fig. 2C) showed two separate clusters within HFA group, further referred to as cluster 1 (n = 12) and cluster 2 (n = 22). Within Cluster 1, an increased variance between data points is visually evident compared to cluster 2.

HFA subjects in Cluster 1 appeared to score higher in NSS total and throughout all included subscales with exact values given in Table 3. Difference in mean scores compared by cluster became significant across all measured NSS (sub)scales except subscale sensory perception.

**Table 3:**
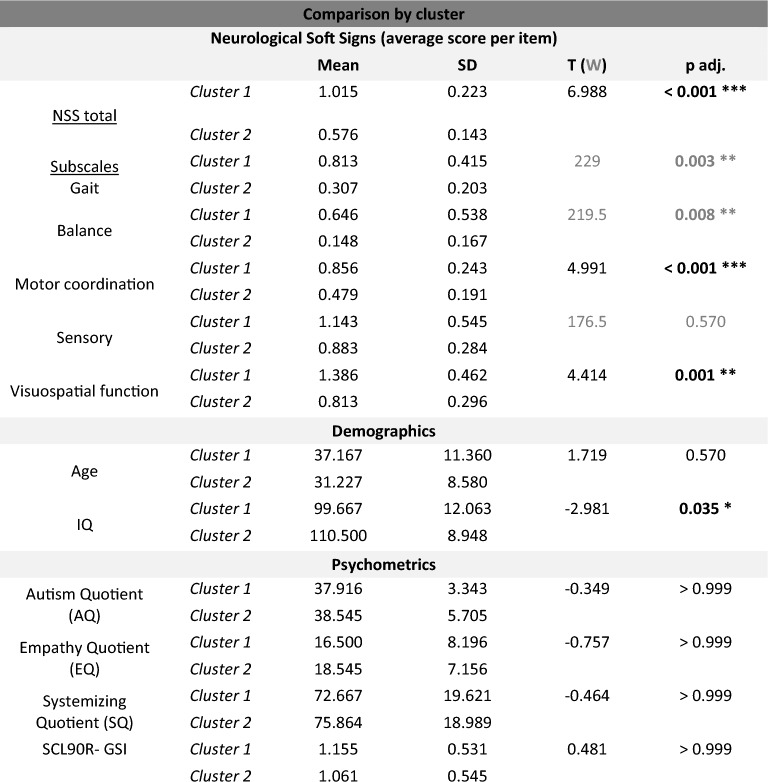
NSS total and subscale scores, demographics and psychometric test results compared by cluster 1 and 2 built upon hierarchical cluster analysis within HFA subgroup (t tests or non-parametric Wilcox tests were applied depending on distribution of data; scales compared by Wilcox test grayed).

Significant values are in bold.

Cluster comparison revealed significant differences on NSS total and all subscales except sensory perception. Moreover, there was a significant difference in mean IQ with lower intelligence level in Cluster 1.

*p < 0.05, **p < 0.01, ***p < 0.001.

Clusters 1 and 2 were further compared for age with no significant difference seen, p adj. = 0.570, but a trend towards higher age in Cluster 1 (Mean age 37.2 ± 11.4 y compared to 31.2 ± 8.6 y in Cluster 2). A significant difference was solely seen in comparison of the intelligence level between clusters. IQ total score measured by WAIS-IV was significantly lower in Cluster 1 (99.7 ± 12.1) compared to Cluster 2 (110.5 ± 8.9), t (33) =− 2.981, p adj. = 0.035. No relevant difference was seen in autism-specific self-rating scale Autism Quotient (AQ) with mean scores of 37.9 ± 3.3 in Cluster 1 and 38.5 ± 5.7 in Cluster 2, p > 0.999. There was also no difference in the Empathy Quotient (EQ), p > 0.999, nor the Systemizing Quotient (SQ), p > 0.999, both displaying core autism-related features. With regard to the global burden of psychological distress measured by SCL90-R (Symptom-Checklist-90-Revised) global severity index (GSI) there was again no difference between the clusters at p > 0.999 with mean GSI 1.155 ± 0.531 in Cluster 1 and 1.061 ± 0.545 in Cluster 2.

As the IQ emerged as prominent factor in the context of cluster analysis, we further performed a correlation analysis between IQ and NSS total and subscale scores to examine which scales are actually influenced by IQ. The correlation analysis revealed significant negative correlations between IQ total and scores on subscales balance, Spearman’s ρ = − 0.218, p = 0.026, and visuospatial function, ρ = − 0.370, p = 0.004, as well as the scores of the entire NSS scale, ρ = − 0.406, p = 0.002.

## Discussion

In this study, using a structured NSS examination, we showed that adults diagnosed with HFA exhibit significantly increased motor coordination deficits compared to healthy controls. In addition, a subgroup of individuals particularly affected by NSS was identified within HFA. This subgroup differed from the less affected in intelligence level but not in autism-typical behavioral traits nor global psychiatric distress.

Our results suggest that adults with HFA differ from healthy individuals by NSS of impaired motor coordination. We see this evidenced by significant differences on the NSS motor coordination subscale between HFA and controls with higher scores in HFA. The NSS subscale motor coordination combines tasks on complex movement sequences of both the lower but mainly the upper extremities (rhythmic foot tapping, opposition of fingers, finger to nose to pencil tasks, alternating hand movements as well as Luria's sequence).

Whether motor deficits are typical in adults with HFA is an open question for which there are at least indications^20^. Recently for example, adults with HFA were shown to display specific signatures in movement timing and balance^7^. While there are only few comparable findings in adults with HFA, our results appear in line to those in children with ASD. In a recent meta-analysis, Wang et al. demonstrated overall significant gross motor deficits in children with ASD, most pronounced, among others, regarding movement of the upper extremities^5^. Contrary, in this study no significant difference was seen on the NSS global scale nor the other motor-associated subscales gait and balance that include further gross motor tasks. Combined, however, the three motor-associated NSS subscales showed a significant group difference with higher average scores in HFA. To this end, we see the thesis of persisting motor impairment in adult HFA reinforced by our data. It should be noted, however, that NSS cannot be considered static as they were frequently shown to vary during the course of psychiatric disease^21^.

The question arises as to the etiology of motor coordination deficits in adults with HFA. Previous studies on autism in children suggested motor deficits to be driven by impaired visual-motor integration^8^ or proprioceptive impairment due to visual fixation problems^22^. Also, Mayoral et al. found higher sensory integration deficits in children with HFA using a structured NSS examination^14^. These theories on the etiology cannot be supported regarding adults by our data. There were no group differences on the subscales regarding sensory perception nor visuospatial ability, although especially the latter subscale includes various visual-motor integration tasks such as copy-figure tests.

Nevertheless, the present results on visuospatial function can be brought in line with previous studies, as the data available is quite heterogeneous. Similar performances between autistic and typical developing individuals in visuospatial processing and visual-constructive ability have been described in children and adolescents^23,24^ as well as adults^25^. Other studies moreover suggested a superior performance of individuals with ASD in visuospatial related tasks. Various theories have emerged to explain altered perception in ASD. The weak central coherence theory suggests a detailed-focused processing bias leading to partial superior results in visuospatial tasks, but with overall conflicting results and specifity remaining unclear^26^. Overall, the vast differences between the test procedures as well as the broad definition of visuospatial or visual-motor ability make comparability to previous work challenging.

Although the results as discussed above can be partly brought in line with previous findings, it must be noted that the promising results regarding NSS examination in adults with HFA could not be reproduced here in their entirety. Both Hirjak et al. and Tani et al. reported global differences between adult HFA and controls with significant higher NSS global scores in HFA^15,16^, which is also described by Mayoral et al. in underage HFA^14^. These findings cannot be supported by our data exhibiting no significant difference in NSS total scores between HFA and sex-, age and IQ-matched controls. On the other hand, a significant motor coordination impairment in HFA can be observed across all different studies using NSS examination, including the present.

The question arises as to why the results of our study differ from the previous ones. It must be considered, that the results are based on different underlying NSS examination procedures. A major difference seems to lie in the results on sensory integration. Mayoral et al. reported significantly higher scores on sensory integration subscale in HFA subjects^14^, whereas the present results suggest a rather opposite effect on the sensory and visuospatial subscale, though in comparison, the applied scales contained same or similar items.

In this regard, interesting results emerged from the hierarchical cluster analysis conducted within the second part of the present study. We showed that there is a subgroup of adults diagnosed with HFA, which is particularly affected by NSS. This subgroup differed significantly from the other subgroup on all NSS subscales except sensory perception. Aside NSS scores, the only distinguishing feature of this group was the significantly lower mean IQ. By contrast, no cluster dependent differences were seen regarding age as evidenced in schizophrenia before^27^. Nor were there cluster dependent differences in sex distribution as might assumed when considering alternating motor performances between sexes shown in autism^28^. Hence, a major difference that could explain the discrepancies with previous studies on NSS in HFA may be the fact that no matching for intelligence level was performed before. For example, Mayoral et al. examined intelligence level but did not match for it, which was accompanied by a substantially lower IQ in the HFA compared with the control cohort. The influence of intelligence level on NSS was previously described in schizophrenia^9^. Also in autism, an influence of intelligence level on global motor function is known, but not by NSS assessment^29,30^. Here, NSS total as well as subscales visuospatial and balance additionally showed a correlation between IQ and NSS scores, supporting the findings based on cluster analysis. Our observation raises the question of whether the NSS scale detects actual neurological deficits or merely intelligence-related difficulties in task implementation or IQ-associated reluctance to perform the tasks. In our opinion, however, this cannot necessarily be concluded, since the mean IQ of the whole HFA group tended towards above average intelligence, whereas cluster 1 had a mean IQ of 99.7, corresponding to the general population average.

We further hypothesized that differentially NSS-affected subgroups of adults with HFA might also show particular autistic or concomitant psychiatric symptomatology as evidenced in children with ASD^5^. Interestingly, our data neither exhibited differences between the clusters in terms of self-rated autistic trait severity, nor regarding general psychological distress. Based on our data no association between NSS and autism core behavioral features nor general psychological distress can be assumed in adults with HFA, which also conflicts with previous studies hinting towards an association of dyspraxia diagnosis and autistic traits in the adult general population^6^.

We acknowledge that the overall sample size remains small but in line with previous studies. In addition, a higher percentage of female participants is obvious, which initially seems unusual given the known differences in prevalence between the sexes. However, the proportion might be well explained by the recruitment procedure. As diagnosis of HFA in females is complicate due to sex differences in phenotype and male-focused diagnostic tools^31^, males with HFA might be diagnosed earlier and so do not present for initial diagnosis in adulthood. On a positive note, the present study thus offers a hitherto scarcely given insight into the often underrepresented group of adult females with HFA.

A potential influence that should be considered, is the application of psychotropic drugs. The difference towards increased psychotropic drug intake in HFA is well explained by the high mental burden in autism and thus in the opinion of the authors reflects the diagnostic reality given.

Moreover, it should be acknowledged, that the psychometric tools used for assessment of autistic and general psychiatric symptoms are self-report scales likely confounded due to perceptional bias. Major limitations also relate to the NSS scale itself and its application. The NSS scale by Gurvits et al.^32^ used in the present study is not established in autism, but broadly comprises other scales like Neurological Evaluation Scale or Heidelberg Scale tested in subjects with autism^14,15^. A general limitation of NSS scales is that the selection of subscales partially appears arbitrary and not evidence based as recently addressed in a meta-analysis of different NSS scales. Due to the overall diversity of NSS scales it seems uncertain to what extent different scales measure the same phenomena, which limits comparability to previous studies^33^. Unlike part of previous NSS studies, the present one lacks statistical evidence on interrater reliability and the conductors of the NSS examination were further not blind for the study hypothesis. It can also be argued that the method chosen here is resource-intensive and does not take into account innovations that result from technological advances. While newer motion capture approaches, as described in Lahnakoski et al. or Budman et al., enable objective, quantitative, technology-based measurements of social and motor behavior^34,35^, the classic NSS assessment has advantages in terms of its established clinical relevance, standardized protocols, and clinical interpretability. Therefore, NSS assessments are likely to remain a valuable complementary tool in the study of motor behavior in neurodevelopmental disorders. In the future, however, it will be advantageous to integrate the classic NSS with contemporary motor observation techniques in order to gain a more comprehensive understanding of motor impairments.

Taking into account the connections between social-cognitive and motor functions mentioned in the introduction with regard to ASD as a “disorder of social interaction”^4^, the data presented here complement an ongoing debate regarding the inclusion of motor impairment in the diagnostic criteria for ASD. The different proposals, however, are so far primarily based on findings in children with ASD. Based on results of large registry studies that demonstrated a high prevalence of motor impairment correlating with autism core behavioral symptoms in autistic children, it has been suggested to implement motor deficits as a diagnostic criterion of ASD or at least to consider motor deficits as an additional specifier of the diagnosis^36,37^. In contrast, other authors argue that impaired motor performance is more likely to be the result of a lack of interest in motor tasks or reduced opportunities to participate in motor-associated activities due to social withdrawal^38^.

We are aware that results from children and adults are not necessarily comparable, yet we consider it important to have this debate for autism diagnosis in adults as well, given the fact that the same criteria are applied for diagnosis.

Based on our data in adults with HFA, we can only support with restrictions the proposal to implement motor impairment as specifier of the diagnosis ASD. We see a supporting aspect in our finding that not all, but a certain subgroup of adults with HFA was particularly affected by NSS (including motor impairment). By contrast, we did not see any association with autism core behavioral features. It remains to be considered that, aside from NSS scores, this subgroup differed only in IQ from the majority HFA group. Intelligence level is a factor already established as a specifier for the diagnosis of ASD. However, the intelligence level of the more NSS affected cluster 1 corresponded to the general population average.

In conclusion, it remains to emphasize the everyday relevance of gross and fine motor skills. Even though, on a positive note, there was no evidence of a correlation between NSS scores and general psychological distress, it might be assumed that adults with HFA experience limitations in everyday life due to motor coordination deficits. We showed that a structured examination of NSS might at least help to identify subjects in need of motor coordination training or intervention. However, we suggest that such structured assessments in adults with HFA should focus on motor skills, rather than sensory integration as evidenced by our data.

## Methods

### Study characteristics

The study included 40 individuals with diagnosis of ICD-10: F84.5 (Asperger’s syndrome), referred to as HFA group since diagnosis of Asperger’s syndrome will be left with ICD-11 (now corresponding to autism spectrum disorder without disorder of intellectual development and with mild or no impairment of functional language). At the time of recruitment, the ICD-10 definition prevailed. We recognize the criticism of the term HFA within the community. However, in our opinion, the term is the most commonly understood for this disorder. Recruitment took place in an outpatient center specializing in the diagnosis of autism spectrum disorders in adulthood. The study protocol adhered to the Declaration of Helsinki and was prospectively reviewed and approved by the ethics committee of Hannover Medical School (approval number 3054–2016). All subjects involved gave written informed consent in participation.

The diagnostic procedure was based on the German guideline for the diagnosis of ASD. A multiprofessional team including two experienced clinicians by minimum performed the diagnostic assessment. The diagnostic interview was based on the Adult Asperger Assessment (AAA) according to Cohen, which builds on the results of the self-rating questionnaires Autism Quotient (AQ) and Empathy Quotient (EQ) in German language^39^. Wechsler Adult Intelligence Scale—IV (WAIS-IV) in German language was used to determine intelligence quotient. HFA subjects between 18 and 65 years of age who fulfilled the diagnostic criteria according to AAA were included. Medical history and concomitant diseases were assessed by interview and questionnaire. Symptom checklist SCL-90-R in German language was used for investigation of subjective impairment by global and specific psychiatric symptoms.

In addition, healthy control subjects were recruited and matched with the subjects for sex (m/f), age (± 5 y), and IQ (± 15 points on WAIS-IV IQ total), since these emerged as common confounding factors in NSS examination^9,27,28^. Apart from the AAA interview, healthy control subjects underwent the same testing procedure.

Subjects with intelligence impairment (IQ total < 70 points) and neurological or other physical disorders potentially associated with major motor, coordination, sensory, or cognitive impairments were excluded both from HFA and control group.

A procedure best described by Greenberg et al.^40^ and based on the NSS scale by Gurvits et al.^32,41^, broadly comprising items of several NSS rating scales and established in several studies based on different psychiatric disorders, was used to assess NSS. A trained psychiatrist or medical student (in total n = 2 investigators involved) administered the test and performed the evaluation based on the given scales. The NSS global scale contains 50 items, each rated according to given criteria on a scale of 0—3 (0 – no deficit, 1 – mild deficit, 2 – moderate deficit, 3 – marked deficit). The individual items were grouped according to Greenberg et al. into the subscales gait, balance, motor coordination, pathological reflexes, sensory, visuospatial function, and others^40^. Supplementary Material S1 provides an overview of the items and their classification into subscales. For comparability, the absolute values for NSS total and subscales were divided by the number of respective items, resulting in average values between 0—3 for each scale. The NSS total scores were calculated by taking into account all the included items in equal proportions. However, for the NSS total and the total motor scale, the calculation was additionally carried out by taking into account the average values of the respective subscales. This was done because the subscales contain different numbers of items, and therefore, if all the individual items are taken into account in equal proportions, there is an imbalance between the domains represented by the subscales.

### Statistical analysis

Sample size estimation and power analysis were performed using G*Power^42^. Based on two prior studies on NSS in HFA^14,15^ with a mean effect size of d = 1.399 across the two studies power analysis suggested a minimum total sample size of N = 30 at a power of 0.95 and a significance level of α = 0.05.

Case–control matching was performed using MedCalc, version 20.114 (MedCalc Software, Ostend, Belgium). SPSS 27 (IBM, Armonk, NY, USA) and R studio 2022.07.1 (Posit Software PBC, MA, USA) were used for statistical analysis and graphical processing.

Depending on distribution of data (assessed by inspection and Shapiro–Wilk test for normality) parametric t-test or non-parametric Mann–Whitney-U or Wilcox-test were applied for comparison of groups. Parts of non-normally distributed data were transformed by calculating square roots. Bonferroni-Holm correction was applied for multiple comparisons.

HFA subjects were further classified by hierarchical cluster analysis using squared Euclidean distance measure and Ward-linkage. HFA subjects previously removed from analysis due to missing match were included for cluster analysis (n = 5, resulting in n total = 34). The resulting clusters were again analyzed for group differences according to above described procedure and supplemented by non-parametric correlation analyses according to Spearman.

### Supplementary Information

Supplementary Information.

## Data Availability

The datasets generated and analyzed during the current study are available from the corresponding author on reasonable request.
