# Supplementary material for: Structured neurological soft signs examination reveals motor coordination deficits in adults diagnosed with high-functioning autism
Source: Sci Rep. 2024 Jul 12;14:16123. doi: 10.1038/s41598-024-66723-5 (PMC11245607; doi:10.1038/s41598-024-66723-5)
Supplement: Supplementary file 1 — Supplementary Information. [file 41598_2024_66723_MOESM1_ESM.docx]

**Supplementary Material**

**Manuscript “Structured neurological soft signs examination reveals motor coordination deficits in adults diagnosed with high-functioning autism” -**

**S1 Neurological Soft Signs by Functional Domain**

*Neurological Soft Signs Examination based on Greenberg, M. S., Wood, N. E., Spring, J. D., Gurvits, T. V., Nagurney, J. T., Zafonte, R. D., & Pitman, R. K. (2015). Pilot Study of Neurological Soft Signs and Depressive and Postconcussive Symptoms During Recovery From Mild Traumatic Brain Injury (mTBI). The Journal of neuropsychiatry and clinical neurosciences, 27(3), 199–205. https://doi.org/10.1176/appi.neuropsych.14050111*

*in () = n items, # = number scoring sheet*

**Gait:**

#1 Normal Walking (1)

#2 Tiptoe-Walking (1)

#3 Heel-Walking (1)

#4 Heel-to-Toe Walking (1)

**Balance:**

# 5 + 6 Standing on One Foot (right/left: 2)

# 7 Romberg Maneuver (with # 8 upper extremity synkinesias) (2)

**Motor:**

# 9 + 10 Foot Tapping (right/left: 2)

# 11 – 14 Opposition of Fingers (right/left + scoring for contralateral synkinesias: 4)

# 15 + 16 Finger-to-Nose (right/left: 2)

# 17 + 18 Finger-to-Nose-to-Pencil (right/left: 2)

# 19 -21 Rapid Alternating Hand Movements (right/left/both: 3)

# 23 – 24 Luria's Sequence (Fist-Edge-Palm) (right/left: 2)

**Reflex:**

# 27 + 28 Palmomental Reflex (right/left: 2)

**Sensory:**

# 29 – 32 Astereognosis (right/left and with/without palpation of objects: 4)

# 33 + 34 Graphesthesia (right/left: 2)

# 35 Extinction (1)

**Visuospatial Function:**

Free Drawing

# 39 Clock (1) # 41 House (1) # 40 Daisy (1) # 53 Face (1)

Copy Figure Test (two sets of stimuli (in total 7))

# 42 a) diamond, b) cross, c) Necker cube, d) smoking pipe

# 43 a) hidden line elimination cube, b) pyramid, c) dissected pyramid

# 44 Assembling Matchstick House (1)

# 50 Map Localizations (Map of Germany) (1)

# 57 + 58 Detection and Recognition of Object Test (degree of difficulty (35 dots per line, 15 dots per line): 2)

# 59 Road-Map Test of Direction Sense (1)

**Other:**

# 52 Rhythm Tapping (1)

# 54 Repeat Tongue Twister (1)
